# The Value of Inflammation Indexes in Predicting Patency of Saphenous Vein Grafts in Patients With Coronary Artery Bypass Graft Surgery

**DOI:** 10.7759/cureus.16646

**Published:** 2021-07-26

**Authors:** Cihan Aydın, Mesut Engin

**Affiliations:** 1 Cardiology, Namık Kemal University Faculty of Medicine Tekirdağ, Tekirdağ, TUR; 2 Cardiovasculer Surgery, Bursa Yuksek Ihtisas Research and Education Hospital, Bursa, TUR

**Keywords:** atherosclerosis, thrombosis, inflammation, coronary artery disease, cabg

## Abstract

Aim:Our study aimed to investigate the predictive values of inflammation markers in predicting postoperative saphenous vein graft patency in patients who underwent coronary artery bypass grafting (CABG).

Method: We retrospectively analyzed 89 patients who undergone CABG, and 49 patients diagnosed with non-critical coronary artery disease (less than ≤50% stenosis) on coronary angiography were included in the study as a control group. Eighty-nine patients who underwent CABG were divided into two groups according to the presence of 50% or more stenosis in saphenous vein grafts. In these three groups of patients, neutrophil to lymphocyte ratio (NLR), derived NLR (dNLR; neutrophils/white blood cells-neutrophils), platelet to lymphocyte ratio (PLR), lymphocyte to monocyte ratio (LMR), systemic inflammation response index (SIRI; neutrophils × monocytes/lymphocytes), systemic inflammation index (SII; platelet × neutrophil/lymphocyte), and the aggregate index of systemic inflammation (AISI; neutrophil × platelet × monocyte/lymphocyte ratio) were calculated from blood tests. The primary endpoint was more than 50% of saphenous vein stenosis or occlusion, and the aim is to predict this saphenous vein graft disease by inflammation indexes.

Results: The groups were similar in terms of the frequency of stroke, diabetes mellitus, and chronic obstructive pulmonary disease. The frequency of heart failure and hypertension was higher in group 2 (p=0.045, p=0.005), respectively. Multivariate logistic regression analysis showed that LMR and NLR levels were independent predictors of saphenous vein graft disease (SVGD; OR: 0.896; 95%CI: 0.465-0.957; P<0.001) , (OR: 0.592; 95%CI: 0.450-0.875; P=0.034, respectively).The cut-off value of the LMR <2.625 was associated with 78.4% sensitivity and 78% specificity to predict saphenous vein graft disease in patients with CABG.

Conclusion: LMR and NLR may be useful predictors for SVGD.

## Introduction

Although endovascular stent technology has advanced day by day, coronary artery bypass grafting (CABG) is still the most effective treatment method in some patient groups. The indications for CABG in patients with stable angina or silent ischemia are; left main coronary artery disease with stenosis >50%, two- or three-vessel disease with stenosis >50% with low ejection fraction.

After CABG, long-term clinical results and recurrent symptoms depend on bypass graft patency and the degree of progression of native coronary artery disease. In vein grafts, faster progression of atherothrombotic occlusive disease causes lower long-term patency rates. Therefore, it has been found in many studies that arterial graft selection increases long-term survival and decreases the frequency of coronary angiographic interventions [1,2]. While the cause of occlusion in vein grafts in the early period (first month) after bypass surgery is thrombotic occlusion, in the late period the reasons are atherosclerosis and neointimal hyperplasia. As it is known, a low grade of continuous inflammation plays a role in the pathogenesis of atherosclerosis. Many inflammation markers have proven to have an important role in predicting cardiovascular mortality [3,4]. In many recent studies, an increase in inflammation risk markers such as neutrophil to lymphocyte ratio (NLR) or platelet to lymphocyte ratio (PLR) after CABG or percutaneous coronary intervention has been found useful in predicting cardiovascular mortality or in-stent restenosis [5].

High NLR and PLR levels have been found in many studies to indicate the severity of coronary artery disease [6,7]. SII, another inflammation marker, is an important new marker in predicting prognosis in various cancer types and elderly patients with acute myocardial infarction [8-10]. Our aim in this study was to predict saphenous vein patency with these new inflammation markers in patients with CABG.

## Materials and methods

Eighty-nine patients who underwent CABG between January 2019 and January 2020 and 49 patients who were diagnosed with the non-critical coronary disease after coronary angiography between these dates were included in our study. Data of these patients were analyzed retrospectively. Control angiography was performed in patients with CABG due to their symptoms approximately one year after the surgical procedure. At the same time, the data of 49 patients diagnosed with non-critical coronary artery disease were retrospectively analyzed.

The study was approved by the institutional ethics committee and conducted in accordance with the Declaration of Helsinki Ethical Principles and Good Clinical Practices.

Coronary artery stenosis below 50% was evaluated as non-critical. Patients who underwent CABG were divided into two groups according to the presence of 50% or more stenosis in saphenous vein grafts. Basic demographic data, coronary angiography reports, clinical history, medications, and test results obtained from blood samples were collected from patients' medical records (Table 1). Systemic inflammation indexes, respectively, NLR (neutrophil/lymphocyte ratio), dNLR (neutrophils/(white blood cells-neutrophils)), PLR (platelet/lymphocyte ratio), LMR (lymphocyte/monocyte ratio), systemic inflammation response index (SIRI (neutrophils × monocytes)/lymphocytes), SII ((neutrophils × platelets)/lymphocytes) and aggregate index of systemic inflammation (AISI; (neutrophils× monocytes ×platelets) /lymphocytes)) were calculated from whole blood assays. Patients with active infectious disease, chronic inflammatory disease, or clinical evidence of cancer were excluded from the study.

**Table 1:** Baseline characteristics of the group. COPD: chronic obstructive pulmonary disease, Ca: calcium, ACE-I: angiotensin-converting enzyme inhibitor, ARB: angiotensin receptor blockers, OAD: oral antidiabetic drug.

Variables	Group 1 (n=49)	Group 2 (n=50)	Group 3 (n=39)	Total (n=138)	p-Value
Age (years)	56.7±10	67.48±8.1	66.4±9.6	62.09±10.5	0.000
Male %	19(38.7%)	31(62%)	32(82.1%)	82(58.7%)	0.000
Female %	30(61.2%)	19(38%)	7(17.9%)	57(41.3%)	0.000
Heart failure %	2(4.08%)	10(20%)	4(10.2%)	16(11.6%)	0.045
COPD %	10(20.4%)	6(12%)	7(17.9%)	23(16.7%)	0.516
Stroke %	2(4.08%)	1(2%)	0(0%)	3(2.2%)	0.425
Hypertension %	37(75.5%)	48(96%)	36(92.3%)	121(87.7%)	0.005
Diabetes mellitus	15(30.6%)	25(50%)	12(30.7%)	52(37.6%)	0.358
Medical treatment					
Beta blocker %	25(51%)	49(98%)	32(82.1%)	106(76.8%)	0.000
Ca-channel blocker %	15(30.6%)	19(38%)	14(35.8%)	48(34.8%)	0.732
ACE-I/ARBs %	33(67.3%)	48(96%)	35(89.7%)	116(84.1%)	0.000
Diuretic %	26(53.06%)	39(78%)	27(69.2%)	92(66.7%)	0.029
OAD %	15(30.6%)	23(46%)	19(48.7%)	57(41.3%)	0.161
Insulin %	5(10.2%)	16(32%)	14(35.8%)	35(25.4%)	0.009

Angiographic analysis

The coronary angiography procedure was performed with the Judkins’ technique in routine standard projections, using appropriate catheters, after obtaining informed consent from each patient. Symptomatic patients diagnosed with stable angina pectoris and patients with ischemia detected in non-invasive tests were included in the coronary angiography procedure.

Coronary angiograms of 138 patients were interpreted by two independent cardiologists who were blinded to the patients' data. The presence of 50% or more stenosis in at least one saphenous graft was considered significant; 50% or less stenosis in native coronary arteries was considered non-critical.

According to the results of coronary angiography, 89 patients who had CABG were divided into two groups; group 3 (N=39) without serious stenosis in saphenous grafts and group 2 (N=50) with severe stenosis in saphenous grafts; 49 patients with non-critical stenosis found in coronary angiography procedures were included in the study as a control group (group 1).

Statistics

All data in the study were analyzed using SPSS 22.0 statistical software (SPSS, Inc., Chicago, IL). The distribution of the data was evaluated with the Kolmogorov-Smirnov test. Continuous variables were expressed as mean ± standard deviation (SD) or median (minimum-maximum), and categorical variables were expressed as a percentage and compared with chi-square or Fischer's exact test. Continuous data conforming to the normal distribution in three independent groups were evaluated with the One-Way Anova test and data not compatible with normal distribution were evaluated with the Kruskal-Wallis test. Mann-Whitney U test was used in the subgroup analysis of the data. The cut-off values for NLR and LMR to predict saphenous vein graft patency were calculated by performing receiver operating characteristic (ROC) curve analysis. Effects of different variables on saphenous vein graft patency were evaluated in univariate analysis for each, and multivariate logistic regression analysis was utilized to predict predictors of saphenous vein graft disease. A p-value of <0.05 was considered statistically significant.

## Results

Baseline characteristics and laboratory results of the patients included in our study are summarized in Tables 1 and 2. In total, 138 patients (mean age 62.09±10.5 years, 82 (58.9%) male) were included. The groups were similar in terms of the frequency of stroke, diabetes mellitus, and chronic obstructive pulmonary disease. The frequency of heart failure and hypertension was higher in group 2 (p=0.045, p=0.005, respectively).

**Table 2:** Laboratory parameters of the groups.

Variables	Group 1 (n=49)	Group 2 (n=50)	Group 3 (n=39)	Total (n=138)	P-value
Glucose (mg/dl)	111(84–309)	122(85–349)	130(77–291)	119(77–349)	0.083
Hemoglobin (g/dl)	13.19±1.49	13.48±1.73	13.3±1.52	13.47±1.51	0.272
Hematocrit %	40.1±5.85	40.6±4.84	41.04±4.37	40.76±4.89	0.236
Platelet count (×10^3^/µL)	227.08±56.4	243.58±54	220.1±77.8	231.41±54.7	0.501
Serum creatinine (mg/dl)	0.81(0.47–1.7)	0.96(0.54–9.67)	1(0.55–5.96)	0.92(0.47–9.67)	0.000
Total cholesterol(mg/dl)	197.2±47.5	215.5±250	178.7±55.5	198.82±155	0.066
High density lipoprotein-cholesterol (mg/dl)	47.1±19.9	42.8±11.3	41.7±14	44.25±15.8	0.219
Low-density lipoprotein cholesterol (mg/dl)	115.7±38.5	103.8±54.6	102.1±44.8	107.72±46.9	0.083
Triglyceride (mg/dl)	150(54–470)	141.9(45–492)	151(40–521)	149.05(40–521)	0.922
High sensitivity c-reactive protein (mg/dl)	2.64(0.3–92)	7.04(0.32–160)	4.1(0.25–372)	4.01(0.25–372)	0.013
White blood cell count (×10^3^/µL)	6.77(4.43–11.74)	7.25(4.79–15.75)	7.45(5.2–16.2)	7.25(4.43–16.23)	0.062
Neutrophil count(×10^3^/µL) (min-max)	4.11(2.07–7.15)	5.18(3.2–13.9)	4.76(3.02–12.4)	4.59(2.07–13.9)	0.000
Lymphocyte count(×10^3^/µL) (min-max)	1.85(1.16–4.17)	1.12(0.54–2.5)	1.84(0.75–3.25)	1.56(0.54–4.17)	0.000
Monocyte count(×10^3^/µL) (min-max)	0.37(0.16–9.5)	0.65(0.22–1.43)	0.57(0.31–1.22)	0.55(0.16–9.5)	0.000
MVP (mean platelet volume)fl	8.8±0.79	10.3±1.2	8.9±0.96	9.4±6.1	0.569
Red cell distribution width %	13.8(11.2–24.2)	14.9(11.6–17.3)	14.6(11.6–25.5)	14.5(11.2–17.3)	0.154
Neutrophil-to-lymphocyte ratio	2.03(0.97–6.6)	2.85(0.71–25)	2.46(1.31–12.2)	2.6(0.71–25)	0.027
Systemic immune inflammation index	457.56(219.1–778.8)	1066.6(458.7–6975.7)	641.8(300–2335.7)	648.57(219.18–6975.74)	0.000
Systemic inflammation response index	0.79(0.25–14.7)	2.72(1.1–28)	1.85(0.58–10.4)	1.56(0.25–28.06)	0.000
Platelet-to-lymphocyte ratio	120.2(54.9–195.7)	220.13(79.1–501.8)	119.4(51.4–280)	133.69(51.41–501.85)	0.000
Lymphocyte-to-monocyte ratio	4.94(0.44–12.92)	1.9(0.5–4.85)	2.91(1.19–6.14)	2.94(0.44–12.94)	0.000
Aggregate index of systemic inflammation	174.04(52.6–4753.5)	691.25(244–76039	403.58(98.54–2185.5)	346.2(52.6–7603)	0.000
Derived neutrophil-to-lymphocyte ratio	1.47(0.76–3.7)	2.33(0.81–11.09)	1.61(0.41–8.26)	1.77(0.41–11.09)	0.000

**Figure 1:**
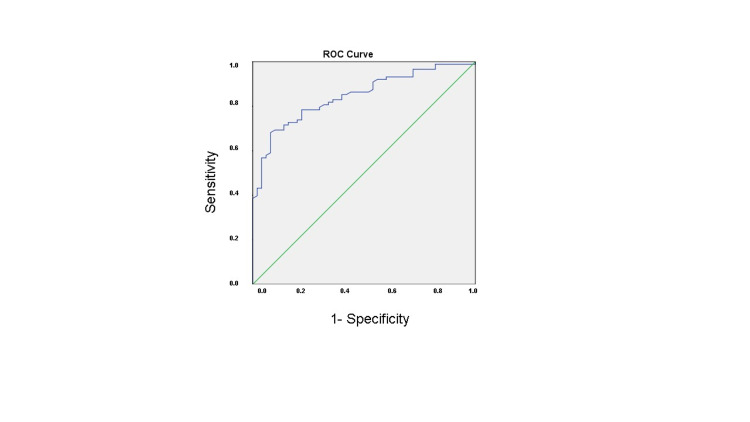
Receiver-operating characteristic curve analysis of lymphocyte-to-monocyte ratio levels for the prediction of saphenous vein graft disease.

**Figure 2:**
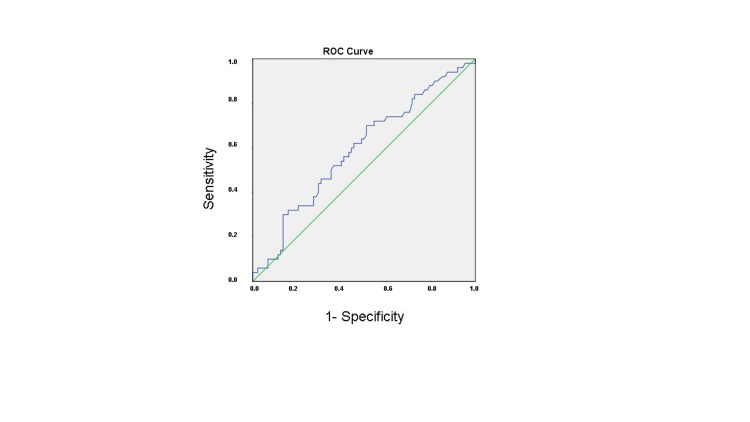
Receiver-operating characteristic curve analysis of neutrophil-to-lymphocyte ratio levels for the prediction of saphenous vein graft disease.

Ca-channel blocker, diuretic, and oral antidiabetic usage rates were similar in all three groups. There was a difference between the usage rates of beta-blocker, ACE-I/ARB, insulin between the three groups (p<0.001, p<0.001, p=0.009, respectively). In terms of biochemical parameters, fasting glucose, hemoglobin, hematocrit, platelet, and white blood cell count, cholesterol, MPV (mean platelet volume), RDW (red cell distribution width), levels were similar between groups. There was a statistically significant difference between the groups in terms of neutrophil, lymphocyte, monocyte counts, and inflammation indexes (SII, SIRI, NLR, PLR, dNLR, LMR, AISI), respectively. In the subgroup analysis, inflammation indexes in groups 2 and 3 were higher than in group 1 except for the PLR (Table 3). Inflammation indexes in group 2 were higher than group 3, except for the NLR. In multivariate logistic regression analysis of the independent predictors, NLR (OR: 0.592; 95%CI: 0.450-0.875; p=0.034) and LMR (OR: 0.896; 95% CI: 0.465-0.957; P<0.001) were all significantly associated with saphenous vein graft disease (Table 4). ROC curve analysis showed that NLR had a sensitivity of 56% and specificity of 56.8% for saphenous vein graft disease in individuals who participated in the study when the cut-off value of NLR was 2.675 (p=0.075). AUC (95%); 0.591 (0.493-0.690; Figure 1 and Table 5). ROC curve analysis also showed that LMR had a sensitivity of 78.4% and specificity of 78% for saphenous vein graft disease when the cut-off value of LMR was <2.625 (p=0.000). AUC (95%); 0.846 (0.782-0.909; Figure 2 and Table 5).

**Table 3:** Subgroup analysis of the indexes.

Variables	Groups 1–2 p-value	Groups 1–3 p-value	Groups 2–3 p-value
Neutrophil-to-lymphocyte ratio	0.001	0.001	0.580
Aggregate index of systemic inflammation	0.000	0.000	0.000
Derived neutrophil-to-lymphocyte ratio	0.000	0.021	0.022
Platelet-to-lymphocyte ratio	0.000	0.132	0.000
Lymphocyte-to-monocyte ratio	0.000	0.000	0.000
Systemic immune inflammation index	0.000	0.001	0.000
Systemic inflammation response index	0.000	0.000	0.002

**Table 4:** Multivariate logistic regression analysis of the independent predictors of late saphenous vein graft disease. CI: confidence interval.

Variables	Odds ratio	95% CI	p-Value
Hypertension	1.226	0.856–2.156	0.194
Diabetes mellitus	1.015	0.880–2.645	0.218
Lymphocyte-to-monocyte ratio	0.896	0.465–0.957	<0.001
Neutrophil-to-lymphocyte ratio	0.592	0.450–0.875	0.034

**Table 5:** ROC curve analysis of NLR and LMR for predicting saphenous vein graft disease. NLR: neutrophil-to-lymphocyte ratio, LMR: lymphocyte-to-monocyte ratio, ROC: receiver operating characteristic, AUC: area under the curve.

Variable	AUC (95%)	Cut off	P-value	Sensitivity%	Specificity%
NLR	0.591 (0.493–0.690)	>2.675	0.075	56	56.8
LMR	0.846 (0.782–0.909)	<2.625	0.000	78.4	78

## Discussion

Currently, CABG is the first option for left main coronary artery lesions and multi-vessel coronary artery disease. Clinical success after CABG depends on saphenous vein graft patency. However, the rapid progression of the atherothrombotic process in vein grafts compared to arterial grafts (left internal mammary artery (LIMA), right internal mammary artery (RIMA), or radial artery) decreases the patency rates of saphenous vein grafts in the long term. While the advantages of venous grafts are easy accessibility, no spasm, and the disadvantages are diameter mismatch, low compliance to arterial pressure, having valves, and easily progressive atherosclerosis. In this current study, we found that indexes such as SII, NLR, obtained from simple blood tests, can predict saphenous vein graft disease in patients undergoing CABG. As well as the selection of the appropriate graft, the location of the anastomosis, excessive pulling, stretching and inflation of the graft are among the causes of early graft occlusion. The 10-year patency rate for SVG is 61% while this rate is 85% for LIMA [11].

Thrombosis at the postoperative first month, neointimal hyperplasia between 1 and 12 months, and atherosclerosis after the twelfth month are the mechanisms responsible for saphenous vein graft occlusion [12]. Inflammation cells such as leukocytes, monocytes, eosinophils, and thrombocytes play a role in the initiation and maintenance of coronary artery disease [13,14].

Especially, monocytes and lymphocytes trigger the process with secreted cytokines and growth hormones such as IL-1, IL-6, and PDGF (platelet-derived growth factor) [15]. Monocytes pass into the subendothelial layer to phagocyte-oxidized LDL (low-density lipoprotein-cholesterol) particles, then transform into foamy cells, and play a role in the formation of the core part of the atheroma plaque. Olivares et al. identified high monocyte and white blood cell levels as an increased risk factor for coronary artery disease [14]. Also, low lymphocyte levels have been associated with poor cardiovascular outcomes in acute coronary syndrome [16]. In our study, we showed that low LMR and high NLR levels are markers that can be used to predict saphenous vein graft patency.

Again, in various studies, low LMR levels were found to be associated with poor endpoints in both critical limb ischemia and in-stent restenosis [17,18]. The effect of LMR value on saphenous vein graft disease was investigated in the study conducted by Oksuz et al. that include 218 patients with CABG. Similar to our study, saphenous vein disease was detected in 51.3% of the patients in the study defined as saphenous vein disease with 50% or more stenosis. In the multivariate analysis, the LMR value (OR: 0.648; 95% CI: 0.469-0.894; p=0.008) was determined as an independent predictor in predicting saphenous vein disease, in addition to the time elapsed after CABG operation and high sensitive CRP values [19]. In our study, we also determined the LMR value as an independent predictor for predicting saphenous vein disease (OR: 0.896, 95% CI: 0.465-0.957, p<0.001).

Four hundred forty-four patients with CABG were included in the retrospective study conducted by Tasoglu et al. In this study, the definition of saphenous vein disease was made similar to our study. At the end of the study, the authors found the NLR value as an independent predictor besides risk factors such as diabetes mellitus and smoking for predicting saphenous vein disease [20].

In our study, we also detected the NLR value as an independent predictor. The SII value is an important inflammatory parameter obtained by multiplying the NLR value with the platelet count. Vasoactive mediators released by platelets may play a role in inflammation and atherogenesis. It has been shown that the MPV value, which is an important indicator of platelet functions, increases the risk of the acute coronary syndrome [21]. In a retrospective study by Kaya and Koza, in which 128 patients were included, the effect of MPV value on saphenous vein disease was investigated. At the end of their studies, the authors showed that the MPV value, which is an important indicator of platelet functions, could be a predictor for saphenous vein disease [22]. In our study, the SII value obtained by multiplying the NLR value with the platelet count was significantly associated with saphenous vein disease.

The importance of SII in predicting coronary stenosis in correlation with fractional flow reserve (FFR) in the native coronary artery and the importance of NLR in predicting saphenous vein graft patency has been demonstrated in previous studies. The anti-inflammatory, antioxidant, antithrombotic, immunomodulatory effects of statin and acetylsalicylic acid in coronary artery disease can be explained by the effect they have on immune cells and cytokine release [23]. In our study, we studied several indexes that could predict late saphenous vein graft occlusion. Among them, although many indexes in subgroup analysis were statistically significant in group 2, we found LMR and NLR as independent predictors in multivariate logistic regression analysis.

Study limitations

In addition to limitations such as the small number of patients included in our study and being retrospective, the patients were not followed up for long-term mortality after angiography. It would be more appropriate to average all the measurements of the patients during their stay in the hospital rather than taking the blood measurements of the patients initially.

## Conclusions

In our study, we demonstrated that LMR and NLR, which are among the new inflammation indexes, are important predictors in determining saphenous graft patency after coronary bypass surgery. The number of repetitive invasive procedures performed on saphenous vein grafts can be reduced with new immunomodulatory treatments available in the future.
